# Patterns of alcohol consumption and risky sexual behavior: a cross-sectional study among Ugandan university students

**DOI:** 10.1186/1471-2458-14-128

**Published:** 2014-02-06

**Authors:** Vikas Choudhry, Anette Agardh, Martin Stafström, Per-Olof Östergren

**Affiliations:** 1 Department of Clinical Sciences, Social Medicine and Global Health, Clinical Research Centre, Lund University, Jan Waldenströms gata 35, House 28, Floor 12, Malmö, Sweden; 2 Department of Pediatrics, Centre for Adolescent Health, Royal Children’s Hospital and Murdoch Children’s Research Institute, University of Melbourne, Melbourne, Victoria, Australia

**Keywords:** University students, Risky sexual behavior, Alcohol use, HIV, Uganda

## Abstract

**Background:**

As reflected in elevated rates of sexually transmitted infections, there is a high prevalence of risky sexual behavior among Ugandan university students. It has been assumed that alcohol contributes to risky sexual behavior. However, perhaps owing to methodological issues, this relationship has found only mixed support in empirical research. The present study analyzes the association between alcohol use and risky sexual behavior at the global, situational, and event level among Uganda university students with sexual experience.

**Methods:**

A cross-sectional survey was carried out in 2010 among 1954 students at Mbarara University of Science and Technology, Uganda, using a self-administered questionnaire. Alcohol use was measured as consumption over the previous 12 months, during situations related to sexual activity and on the most recent occasion of sexual intercourse. Risky sexual behavior was defined as having two or more sexual partners in the previous 12 months or inconsistent condom use with new partners. Bivariate and multivariate logistic regression was performed to analyze the association between alcohol use and risky sexual behavior separately for males and females.

**Results:**

Even after adjusting for confounders, the odds ratio (OR) of having two or more sexual partners in the past year indicated a statistically significant association with alcohol use on all levels (global, situational, and event) for both males and females. The ORs of inconsistent condom use with a new partner were significant for males who often consumed alcohol in relation to sexual activity—even after adjusting for potential confounders (OR, 1.75; confidence interval, 1.01–3.08). The risk of inconsistent condom use with a new partner was twice as high for females who often consumed alcohol in relation to sexual activity, although this association was not statistically significant.

**Conclusions:**

The study supports previous research that alcohol consumption is associated with having multiple sexual partners. Inconsistent condom use was associated with the situational use of alcohol in relation to sexual activity and was similar for both genders. Interventions to reduce alcohol-related risky sexual behavior should target both male and female drinkers, particularly subgroups of students, who often consume alcohol in relation to sexual activity.

## Background

After the initial success of the massive decline in the early 1990s to 2000s, HIV prevalence rates now seem to have risen again in Uganda. HIV prevalence among women and men aged 15–49 years increased from 6.4% in the 2004–05 HIV/AIDS—Behavioral Survey to 7.3% in the 2011 Uganda AIDS indicator survey and according to the latter survey, 3.7% of young people aged 15–24 are HIV positive [1]. As in other parts of sub-Saharan Africa, young people in Uganda generally show a high prevalence of unsafe sexual behavior [2]. Although Ugandan adolescents now appear to be delaying their sexual debut, the majority become sexually active by the age of 18 and are likely to engage in risky sexual behavior, such as unprotected sexual intercourse and multiple sexual partnerships [3-6].

In 2010, a survey conducted at six universities in Uganda estimated the HIV prevalence among students there to be 1.2%, which was lower than the national average [7]. However, the survey also indicated that the prevalence of all sexually transmitted infections (STIs) over the previous 12 months was 10%, which was somewhat higher than the estimated national average for that period [7]. Research has indicated that sexual activity on Uganda’s campuses is often in the form of multiple sexual partnerships, temporary relationships, transactional sex [7], and coercive sex [8]. Efforts to curb the spread of HIV among young people in Uganda have mainly focused on the general population of young people or the general population of most-at-risk populations, instead of focusing on specific groups of young individuals [7]. Moreover, there is a lack of information about STIs, including HIV prevalence and contraceptive use within such subpopulations as university students, since routine sources of data (such as HIV surveillance and national population-based AIDS indicator surveys) do not provide this kind of detail [7]. Research is necessary to identify the risk factors in these subpopulations and generate evidence to guide interventions toward preventing the transmission of STIs, including HIV infection.

An alcohol research project called the multinational project on Gender, Alcohol and Culture: an International Study noted that in Uganda, 6% (1936) of the females and 2% (595) of the males sampled between the ages of 12 and 24 reported that they or their partners had been drinking the last time they had sexual intercourse in the 12 months preceding the survey [9]. The contribution of alcohol to risky sexual behavior (multiple sexual partners or unprotected sex) has been implicated in the spread of STIs [10], including HIV [11-14].

According to the latest global report on alcohol use, Ugandans are the highest per capita consumers of alcohol in Africa [15]. The large number of abstainers indicates that those people who do drink tend to consume substantial amounts of alcohol [15]. Young people in Uganda seem to follow a similar pattern of alcohol consumption to that of the general population. A study carried out at Mbarara University in Uganda indicated that almost half of the students had consumed alcohol in the previous 12 months, and a quarter of them had engaged in heavy episodic drinking [16]. Alcohol is easily available to students at Uganda’s universities, and according to local reports alcohol consumption is considerable, particularly during celebrations [17]. In addition, university authorities have acknowledged that alcohol is the main factor in declining academic performance, mental health problems, rape, and violence on and off campus [17].

Studies have shown that consuming alcohol may lead to sexual risk-taking through the agency of behavioral and biological mechanisms. According to the alcohol myopia theory [18], alcohol disinhibits behavior owing to its pharmacological effects on cognitive capacities. Thus, proximal and simple cues that instigate behavior (e.g., sexual arousal) continue to be processed, whereas more distal and complex cues that would ordinarily inhibit behavior (e.g., the possibility of contracting an STI or an unplanned pregnancy) are no longer adequately processed. By contrast, the expectancy model proposes that an individual’s behavior after drinking is driven by preexisting beliefs and expectations about possible effects of alcohol in the manner of a self-fulfilling prophecy [19]. These individual expectations about alcohol may themselves result in lower risk perceptions, unsafe intentions, and various forms of risky sexual behavior [20]. Researchers have used confounding variables, such as personality factors of sensation seeking or compulsivity, in attempting to explain the association between alcohol and STIs, including HIV and AIDS [21,22].

The association between alcohol use and risky sex has received mixed support in empirical studies. For the most part, the evidence has been based on studies that use measures of generalized alcohol consumption and various indicators of unsafe sexual behavior by means of cross-sectional methodological approaches. Our review of the literature supports the notion that alcohol use is related to sexual risk-taking when it involves multiple sexual partnerships [23-26], but there are mixed results regarding the association between alcohol consumption and inconsistent condom use [27].

The association between alcohol consumption and risky sexual behavior has been studied at three levels of analysis: alcohol use in general as a global association; the use of alcohol in relation to sexual activity as a situational association; and the use of alcohol in relation to a sexual event, such as the first or most recent occasion of sexual intercourse, as an event-level association. Most studies have addressed the issue of general alcohol consumption and its global associations, i.e., whether the extent to which an individual engages in one type of behavior, such as frequent alcohol use, is associated with other types of risky behavior, such as inconsistent condom use [28]. These studies have often been criticized for their methodological limitations in not being able to show any form of temporality or causality [25]. The second level of association indicated above—situational—occurs when an individual who consumes alcohol in a specific situation is also more likely to engage in sexual intercourse in that setting [27]. Studies at this level have been criticized for not being sufficiently robust methodologically to demonstrate that alcohol consumption and high-risk sexual behavior occur at the same time [23]. Finally, event-level studies have typically analyzed data concerning a particular type of behavior occurring on a specific occasion, e.g., consumption of alcohol on the most recent occasion of intercourse [24]. Studies have been unable to reliably link the inconsistent use of condoms to sexual situations in which alcohol consumption is also involved—either at the situational level [24] or with regard to a specific intercourse event [23,27]. However, to the best of our knowledge, very few studies have applied all three levels of analysis within the same population.

Despite the imminent threat of STIs, including HIV and AIDS, the issue of alcohol use and sexual risk-taking among university students in Uganda has largely been unaddressed. In light of this situation and since there is concern about the HIV status of young people at universities, it is important to gain further insight into the relationship between alcohol and risky sexual behavior. The current study aims to analyze the association between alcohol use and risky sexual behavior at the global, situational, and event level among Ugandan university students with sexual experience.

## Methods

### Population and setting

The data for this study were derived from a cross-sectional survey conducted in 2010 at Mbarara University of Science and Technology (MUST) in southwest Uganda. All enrolled undergraduate students at various faculties in MUST were invited to participate in the survey. Of the 2706 enrolled undergraduate students at the university, 1954 completed the survey (response rate 72%). A total of 1179 (60%) reported having had sexual experience. Our analysis was carried out on those 1179 individuals since we wished to analyze the association between alcohol and risky sexual behavior.

The present study was a follow-up to a survey conducted at MUST in 2005, using the same questionnaire [29]. The questionnaire was administered in English and consisted of 132 items; it addressed alcohol and other substance use, social capital, self-rated health in general and mental health, sexuality, and sexual relations. In addition, there were questions on basic sociodemographic characteristics, such as age, sex, area of growing up, religious affiliations, and the role of religion in the family life of the individual during childhood and adolescence. The questionnaire was developed in 2005 based on focus group discussions held with students in the same university [30] and instruments validated in other studies [8,29,31].

For the present study, the survey took place in lecture halls at MUST, with a member of the research team present. Before distributing the questionnaire, a consent form was circulated, describing the purpose of the study; students were asked to sign the form if they agreed to participate. Participation in the survey was voluntary, and anonymity was assured and maintained. The contact details of the project’s principal investigator and research assistant were provided in case students had particular questions. The Institutional Review Committee at MUST approved the questionnaire and study design.

### Study measures

#### Sociodemographic variables

##### Age

Age was applied as a dichotomized variable and categorized as ≤22 or >22 years. The cutoff was based on the median in the sample distribution.

##### Role of religion

This variable was based on the question “What role did religion play in your family when you were growing up?” The response alternatives were dichotomized: “religion played a major role” and “religion was relatively important” were coded as “major role of religion when growing up;” “religion was not so important” and “religion was not important at all” were coded as “minor role.”

##### Area of growing up

This question sought to determine whether students grew up in an urban, semi-urban, or rural setting. Combining urban and semi-urban into one category and rural into another dichotomized the variable.

#### Exposure variables related to alcohol

##### Alcohol use over the past 12 months

This variable was based on the question “How often have you consumed alcohol during the past 12 months?” The alternatives to this question were as follows: (1) four times a week or more; (2) two to three times a week; (3) three to four times a month; (4) once a month or seldom; and (5) never. The first three alternatives were coded as “frequent alcohol use” whereas the fourth alternative was categorized as “seldom alcohol use.” Those who responded with the fifth alternative were classed as abstainers.

##### Alcohol use in relation to sexual activity

This variable was based on the question “How often do you drink alcohol before having sexual intercourse?” with the options being as follows: (1) always or almost always; (2) over 50% of the time; (3) around 50% of the time; (4) less than 25% of the time; and (5) almost never or never. This variable was then dichotomized by combining the first three alternatives as “alcohol used in relation to sexual activity” and the last two as “non-use of alcohol in relation to sexual activity.”

##### Alcohol use on the last occasion of sexual intercourse

This variable was based on the question “Had you been drinking any alcohol the last time you had sexual intercourse?” The alternatives were defined as yes or no.

#### Outcome variables related to sexual behavior

##### Multiple sexual partnerships

Students were asked “How many sexual partners have you had in the last 12 months?” This open-ended question was later dichotomized into fewer than two or two or more sexual partners; the latter was categorized as having “multiple sexual partnerships.”

##### Inconsistent condom use with a new partner

This variable was based on the question “How often do you use a condom with a new sexual partner?” The alternatives to this question were as follows: (1) always; (2) often; (3) sometimes; (4) never; and (5) the question does not apply to me. The alternatives were then dichotomized as “consistent condom use with a new partner” only for students who answered “always” (alternative 1); the students who gave other alternatives were categorized as “inconsistent condom use.”

Our selection of outcome variables and cutoff points within them was based on international indicators previously used in demographic health surveys, AIDS indicator surveys, and other studies in Uganda [1,3,7].

### Statistical analysis

Statistical analysis was conducted using IBM- SPSS version 20.0. Confidence intervals (CIs) were calculated at the 95% level to estimate statistical significance. Chi-square tests were performed to examine differences in the proportions of males and females for both dependent and independent variables. We performed bivariate and multivariate logistic regression analyses to assess the associations between alcohol use and risky sexual behavior variables. The confounders in the study were chosen based on the literature. A stepwise adjustment for age, area of growing up, and role of religion when growing up was made when carrying out the multivariate regression analysis. The same confounders were used for analyzing the association between different alcohol exposure variables and both outcomes of risky sexual behavior used in the study. The adjusted odds ratio (OR) changed very little at each step for main alcohol exposure; hence, the final model is shown adjusted for age and residential area and role of religion when growing up. Separate analyses were performed for males and females to detect gender patterns in the associations. The missing values represent individuals who gave incomplete answers for at least one of the variables included in the multivariable logistic regression analysis. Since we decided not to use any imputation method for missing values, those cases were not included in the logistic regression analysis.

## Results

The prevalence of various sociodemographic characteristics, patterns of alcohol use in general and in combination with sexual activity, and sexual behavior for males and females with sexual experience have been presented in Table 1. The mean age in the study sample was 23 years. As indicated in Table 1, frequent alcohol consumption over the past 12 months was more common among male students. Among those who drank, approximately 27% had consumed alcohol when they last had sexual intercourse. There was a significant difference in the proportions of males and females engaging in this behavior. About 20% of those who drank reported using alcohol in conjunction with sexual activity, with a significantly higher proportion of them being males. Approximately 33% of all students reported having had two or more sexual partners in the last 12 months. Again, this was more prevalent among males. Among the women, around 50% reported inconsistent condom use with a new partner, whereas only about 37% of the males reported the same.

**Table 1:** Descriptive and chi-square test results of gender differences across sociodemographic factors, alcohol use, and sexual behavior in a sample of Ugandan university students with sexual experience

**Variables**	**Characteristics**	**All (N = 1179)**	**Females (N = 486)**	**Males (N = 693)**	**Chi-square**
		**n (%)**	**n (%)**	**n (%)**	**(p-value)**
Age	Younger (≤ 22 years)	608 (51.6)	215 (44.0)	393 (56.9)	<.001
	Older (> 22 years)	571 (48.4)	271 (56.0)	300 (43.1)	
Role of religion while growing up	Major role of religion while growing up	736 (62.5)	334 (68.5)	402 (58.2)	<.001
	Minor role of religion while growing up	443 (37.5)	154 (31.5)	289 (41.8)	
Area of growing up	Urban	610 (51.7)	272 (56.0)	338 (48.8)	.009
	Rural	569 (48.3)	214 (44.0)	355 (51.2)	
Alcohol use in the last 12 months	Abstainers of alcohol	520 (46.8)	240 (54.3)	280 (41.9)	<.001
	Seldom users of alcohol in last 12 months	450 (40.5)	163 (36.9)	287 (43)	
	Frequent users of alcohol in last 12 months	140 (12.6)	39 (8.8)	101 (15.1)	
	Missing	69	44	25	
Alcohol use in relation to sexual activity*	Non-Use of alcohol in relation to sexual activity	391 (79)	146 (83.4)	245 (76.6)	.002
	Use of alcohol in relation to sexual activity	104 (21)	29 (16.6)	75 (23.4)	
	Missing	95	27	68	
Alcohol use at latest occasion of sexual intercourse*	Non-Use of alcohol at latest occasion of sexual intercourse	360 (72.9)	133 (76.9)	227 (70.7)	.001
	Use of alcohol at latest occasion of sexual intercourse	134 (27.1)	40 (23.1)	94 (29.3)	
	Missing	96	29	67	
Condom use with new partner	Consistent use of condom with new partner	603 (57.8)	216 (50.8)	387 (62.6)	<.001
	Inconsistent condom use with new partner	440 (42.2)	209 (49.2)	231 (37.4)	
	Missing	136	61	75	
Number of sexual partners in past 12 months	0-1 sexual partner in last 12 months	680 (66.4)	324 (77.5)	356 (58.5)	<.001
	2 or more sexual partners in last 12 months	344 (33.6)	94 (22.4)	250 (41.3)	
	Missing	155	68	87	

*Abstainers from alcohol over 12 months have been excluded from this analysis.

Table 2 presents the bivariate logistic regression analysis between sociodemographic variables, alcohol use in general, drinking often in relation to sexual activity, and drinking at the time of last intercourse and sexual behavior. Alcohol consumption in the last 12 months, alcohol use in relation to sexual activity and alcohol use on the last occasion of sexual intercourse was associated with having had two or more sexual partners. Students who often-consumed alcohol in relation to sexual activity were found to have an almost 1.5-fold higher risk for inconsistent condom use with a new partner (OR _crude_, 1.64; CI, 1.05–2.57).

**Table 2:** **Bivariate associations (crude odds ratio [OR **_**crude**_**], 95% confidence intervals [CI]) between sociodemographic factors and the use of alcohol with risky sexual behavior in a sex-stratified sample of Ugandan university students with sexual experience**

**Variables**	**Characteristics**	**Multiple sexual partnerships**			**Inconsistent condom use with new partner**		
		**All**	**Females**	**Males**	**All**	**Females**	**Males**
Age	Older (> 22 years)	**.75 (.58–.98)**	.78 (.49–1.20)	.84 (.60–1.17)	1.12 (.87–1.44)	1.07 (.73–1.58)	1.04 (.75–1.46)
Role of religion while growing up	Minor role of religion while growing up	**1.37 (1.05–1.78)**	1.37 (.85–2.20)	1.23 (.89–1.70)	.97 (.75–1.25)	.86 (.57–1.30)	1.15 (.83–1.61)
Area of growing up	Rural	.80 (.62–1.04)	.70 (.44–1.12)	.77 (.56–1.06)	**1.67 (1.30–2.14)**	**1.67 (1.14–2.46)**	**1.81 (1.30–2.52)**
Alcohol use in the last 12 months	Seldom users of alcohol in last 12 months	**1.55 (1.15–2.07)**	1.59 (.96–2.67)	1.36 (.95–1.98)	.78 (.60–1.02)	.76 (.50–1.15)	.85 (.60–1.23)
	Frequent users of alcohol in last 12 months	**3.89 (2.57–5.86)**	**3.56 (1.62–7.82)**	**3.34 (2.03–5.50)**	1.03 (.70–1.52)	1.00 (.49–2.06)	1.20 (.74–1.93)
Alcohol use in relation to sexual activity*	Use of alcohol in relation to sexual activity*	**3.68 (2.26–5.98)**	**5.10 (1.97–13.18)**	**3.01 (1.70–5.34)**	**1.64 (1.05–2.57)**	1.91 (.80–4.56)	1.66 (.97–2.83)
Alcohol use at latest occasion of sexual intercourse*	Use of alcohol at latest occasion of sexual intercourse*	**2.94 (1.90–4.53)**	**4.45 (1.97–10.06)**	**2.39 (1.41–3.94)**	1.32 (.7–2.00)	1.68 (.80–3.56)	1.23 (.75–2.03)

*Abstainers of alcohol during the last 12 months have been excluded from this analysis.

The bold font indicates statistical significance at p<.05.

Table 3 shows the results of multivariate logistic regression analysis performed to account for possible confounding in the association between alcohol use over the previous 12 months and risky sexual behavior. In the adjusted analysis, among students who frequently consumed alcohol in the last 12 months, both females (OR _adjusted_, 4.08; CI, 1.77–9.41) and males (OR _adjusted_, 3.55; CI, 2.12–5.96) were at higher risk of having had more than one partner during that period than students who abstained from alcohol use.

**Table 3:** **Multivariate associations (adjusted odds ratio [OR **_**adjusted**_**], 95% confidence intervals [CI]) between the use of alcohol over the past 12 months with risky sexual behavior in a sex-stratified sample of Ugandan university students with sexual experience**

**Characteristics**	**Multiple sexual partnerships**			**Inconsistent condom use with new partner**		
	**All**	**Females**	**Males**	**All**	**Females**	**Males**
Abstainers	1 (ref)	1 (ref)	1 (ref)	1 (ref)	1 (ref)	1 (ref)
Seldom users of alcohol	**1.48 (1.10–2.01)**	1.50 (.89–2.54)	1.33 (.92–1.95)	.80 (.60–1.07)	.83 (.54–1.30)	.85 (.58–1.25)
Frequent users of alcohol	**4.16 (2.71–6.40)**	**4.08 (1.77–9.41)**	**3.55 (2.12–5.96)**	1.11 (.74–1.67)	1.31 (.60–2.86)	1.24 (.75–2.04)
Older (> 22 years)	**.68 (.51–.90)**	.65 (.40–1.08)	.77 (.54–1.10)	1.25 (.96–1.64)	1.12 (.74–1.72)	1.21 (.85–1.72)
Rural residence while growing up	.80 (.60–1.07)	.74 (.44–1.24)	.78 (.55–1.11)	**1.81 (1.39–2.37)**	**1.79 (1.16–2.73)**	**1.96 (1.38–2.8)**
Minor role of religion while growing up	1.31 (.98–1.74)	1.14 (.69–1.92)	1.29 (.90–1.82)	1.04 (.80–1.37)	.90 (.57–1.40)	1.25 (.89–1.78)

The bold font indicates statistical significance at p<.05.

Table 4 presents the adjusted relationships between alcohol use in relation to sexual activity and risky sexual behavior among the students. After adjustment for confounders, male (OR _adjusted_, 1.75; CI, 1.01–3.08) and female students (OR _adjusted_, 2.35; CI, 0.91–6.08) who often consumed alcohol in relation to sexual activity were found to have a higher risk of inconsistent condom use with a new partner.

**Table 4:** **Multivariate associations (adjusted odds ratio [OR **_**adjusted**_**], 95% confidence intervals [CI]) between sociodemographic factors and the use of alcohol in relation to sexual activity with risky sexual behavior in a sex-stratified sample of Ugandan university students with sexual experience**

**Characteristics**	**Multiple sexual partnerships**			**Inconsistent condom use with new partner**		
	**All**	**Females**	**Males**	**All**	**Females**	**Males**
Non–Use of alcohol in relation to sexual activity*	1 (ref)	1 (ref)	1 (ref)	1 (ref)	1 (ref)	1 (ref)
Use of alcohol in relation to sexual activity*	**4.18 (2.50–7.00)**	**5.65 (2.09–15.23)**	**3.47 (1.90–6.35)**	**1.78 (1.11–2.86)**	**2.35 (.91–6.08)**	**1.75 (1.01–3.08)**
Older (> 22 years)	.90 (.60–1.36)	1.02 (.50–2.12)	.90 (.54–1.47)	1.32 (.90–1.96)	1.58 (.81–3.08)	1.12 (.68–1.86)
Rural residence while growing up	1.01 (.68–1.53)	1.32 (.62–2.80)	.84 (.52–1.37)	**2.18 (1.47–3.25)**	**2.68 (1.33–5.40)**	**2.20 (1.34–3.60)**
Minor role of religion while growing up	1.50 (.99–2.22)	1.51 (.73–3.14)	1.48 (.90–2.42)	.98 (.65–1.44)	1.05 (.54–2.05)	1.00 (.61–1.65)

*Abstainers of alcohol during the last 12 months have been excluded from this analysis.

The bold font indicates statistical significance at p<.05.

Table 5 shows the relationship between alcohol use on the last occasion of sexual intercourse and risky sexual behavior among the students in a multivariate model. Both male (OR _adjusted_, 2.52; CI, 1.48–4.30) and female students (OR _adjusted_, 5.47; CI, 2.30–12.97) who had consumed alcohol at the time of last sexual intercourse were found to have a higher risk of multiple sexual partnerships over the previous 12 months.

**Table 5:** **Multivariate associations (adjusted odds ratio [OR **_**adjusted**_**], 95% confidence intervals [CI]) between sociodemographic factors and the use of alcohol on the latest occasion of sexual intercourse with risky sexual behavior in a sex-stratified sample of Ugandan university students with sexual experience**

**Characteristics**	**Multiple sexual partnerships**			**Inconsistent condom use with new partner**		
	**All**	**Females**	**Males**	**All**	**Females**	**Males**
Non–Use of alcohol at latest occasion of sexual intercourse*	1 (ref)	1 (ref)	1 (ref)	1 (ref)	1 (ref)	1 (ref)
Use of alcohol at latest occasion of sexual intercourse*	**3.25 (2.07–5.10)**	**5.47 (2.30–12.97)**	**2.52 (1.48–4.30)**	1.50 (.98–2.32)	2.23 (.99–5.05)	1.35 (.80–2.27)
Older (> 22 years)	.88 (.59–1.32)	.94 (.45–2.00)	.88 (.54–1.45)	1.45 (.97–2.13)	1.75 (.90–3.44)	1.18 (.71–1.95)
Rural residence while growing up	.99 (.66–1.48)	1.56 (.72–3.42)	.75 (.46–1.22)	**2.04 (1.40–3.02)**	**2.57 (1.28–5.15)**	**1.97 (1.20–3.20)**
Minor role of religion while growing up	**1.62 (1.08–2.42)**	1.70 (.80–3.66)	1.58 (.97–2.58)	1.02 (.69–1.50)	.94 (.48–1.85)	1.13 (.70–1.85)

*Abstainers of alcohol during the last 12 months have been excluded from this analysis.

The bold font indicates statistical significance at p<.05.

## Discussion

The current study analyzed the association between alcohol use and risky sexual behavior at the global, situational, and event level among Ugandan university students with sexual experience. Among MUST students, we found a higher percentage of males who consumed alcohol in general and in relation to sexual activity. As elsewhere in the world, men in Uganda are more likely than women to consume alcohol and drink more before sex [31,32]. Our study found a significant association between alcohol use in general, alcohol consumption in relation to sexual activity, and alcohol use on the last occasion of sexual intercourse with having multiple sexual partners. In addition, students who often consumed alcohol in relation to sexual activity were also at a higher risk of inconsistently using condoms with a new partner. We found similar patterns of associations among both male and female students, although existing social and cultural beliefs may impact the prevalence and strength of the gender-based association between alcohol consumption and risky sexual behavior.

As elsewhere in the world, university campuses in Africa are spaces of sexual exploration [33]. Our study found that 33% of the sample engaged in multiple sexual partnerships, with almost 41% of the males having had more than one sexual partner in the previous 12 months as compared with 23% of the females. However, our study did indicate that both male and female university students had a higher risk of having multiple sexual partnerships after alcohol use of any kind. Our findings are congruent with previous research, which indicated a greater likelihood of multiple sexual partnerships in association with frequent drinking—either in general or in relation to sexual activity—with both sexes, with different races, and in the general population [13,25,34,35]. This result is consistent with studies conducted on university student populations in different countries [2,36,37].

Although we did not examine the nature of the relationship in cases of multiple sexual partners, we speculate that many of them may have been temporary in nature; this is in light of a study conducted in six Ugandan universities [7], which found that as many as 73% of all relationships that developed in such universities were temporary. In addition, some of those temporary sexual relationships at university may have involved partner overlaps, which facilitates STI transmission in this subgroup. Multiple sexual partnerships, especially those that are concurrent, have been implicated in the increased rates of HIV in the latest estimates by the Uganda AIDS Commission [1]. In Uganda and elsewhere in Africa, relationships in university students have been described in terms of prevalent multiple sexual partnerships, including transactional sex with an older partner at the same time as having a romantic partner on campus. This is especially true of young female students [7,38]. Earlier qualitative studies have indicated that these intergenerational and transactional sexual partnerships, which are thought to play an important role in the transmission of HIV [39], are often initiated in drinking establishments [40].

In our study, any alcohol use—whether seldom or frequent over the previous 12 months or in relation to sexual activity or use on the last occasion of sexual intercourse—was associated with increased risk of having had two or more sexual partners during the same period. This relationship perhaps supports the disinhibitory effect of alcohol combined with certain social environments at university, such as parties and drinking alcohol at bars, which also facilitate the meeting of new sex partners. The association between alcohol consumption in relation to sexual activity and having multiple sex partners may perhaps also be explained by the alcohol expectancy theory [41]. This theory holds that individuals who think drinking alcohol will cause them to become less nervous, more sexually uninhibited, and thus at greater ease in potential sexual situations are more likely to drink before a possible sexual encounter in certain social situations, such as on a date, at a party, or at a bar [42].

Our study found that students who often consumed alcohol in relation to sexual activity were at increased risk of inconsistent condom use with new partners. However, the lack of a relationship between risky alcohol consumption in general or with the specific event of the last sexual intercourse and inconsistent condom use with new partners suggests that disinhibition or the social situational effect may play only a minor role in this context. Conversely, the individual personality trait often referred to as sensation seeking, which includes such risky sexual behavior as inconsistent condom use, could be an attribute of students who often consume alcohol in relation to sexual activity [25,27,43]. Previous research has also demonstrated that individuals who engage in one form of risky behavior often tend to indulge in other forms [2]. Our findings are consistent with those of recent studies, showing that men and women who reported alcohol use in sexual situations were likely to engage in unprotected sex [35,44]. Inconsistent condom use among these students in combination with multiple sexual partnerships that are often temporary and concurrent in nature increases the risk of HIV transmission among this subgroup of students.

We also found an association between rural residence when growing up and inconsistent condom use among both male and female university students. Perhaps the sudden freedom from parental control and exposure to a more liberal urban environment influences sexual risk-taking of this sort. Though it is difficult to clarify this phenomenon on the basis of the present study, future research could examine students who have shifted to an urban university environment from a traditional rural upbringing to see how that affects their sexual behavior and alcohol consumption.

Public health interventions, including messages about alcohol use, could strengthen preexisting beliefs about the possibility of alcohol leading to risky sexual encounters: care should be taken that young people are not given an excuse for engaging in unprotected sex [24]. Interventions should be pragmatic and target both male and female drinkers. These interventions should emphasize that drinking carries with it the increased likelihood of having sex with someone you do not know and that such encounters carry a high risk of exposure to STIs and HIV. Policies directed at limiting the availability of alcohol on university campuses and programs targeting responsible consumption of alcohol on campuses should also be stressed.

### Strengths and limitations

One strength of this study is that the analysis includes all three levels of associations: global, situational, and event. Another strength is that it included the entire student population within a university and had a high response rate, thereby reducing the issue of selection bias. However, we recognize that our study sample of students at one university is not representative of all young people in Uganda.

The data were gathered through a questionnaire with retrospective self-reporting. As a result, it may have suffered from recall bias. Previous research on this issue has suggested that reporting biases that occur on the basis of social desirability tend to be under-reported rather than over-reported [45].

Another limitation of a cross-sectional study is that it cannot identify the causal mechanisms with some of the observed associations. Hence, we cannot be certain that alcohol influences sexual risk-taking or that individuals with multiple risk-taking tendencies are also likely to consume more alcohol and participate in sexual risk-taking behavior. We sought to mitigate the lack of temporality between exposure and outcome by analyzing the use of alcohol consumption in relation to sexual activity at both the situational and event level and determining the association with risky sexual behavior.

This study provides little information about peer norms, personality factors of sensation seeking, coping mechanisms, or social context factors that might constitute a possible third-variable explanation of alcohol consumption and risky sexual behavior among young people; this area has been examined in previous studies on young people and general populations [25,27,43,46]. Although we obtained data on event-specific use of alcohol and the sexual behavior of particular individuals, we were unable to acquire partner data, which would have been useful in interpreting the relationship between alcohol and sexual behavior, especially condom use and gender differences. It would also have been helpful to incorporate details of the partner’s alcohol consumption. Diary studies are a specific method for measuring alcohol use as well as risky sexual behavior. They could be useful in assessing this question, although the method is difficult to apply with young people in a university environment or with national studies.

## Conclusions

In our sample of university students, alcohol consumption was found to be associated with having multiple sexual partners and inconsistent condom use with new partners among students who often used alcohol in relation to sexual activity. The relationship between alcohol use and risky sexual behavior is complex, and it may be influenced by a combination of social, physiological, and individual personality traits. Our findings suggest that there is a need to focus on the role of alcohol in risky sexual behavior in the design and implementation of HIV-prevention programs for university students. Such interventions to reduce alcohol-related risky sexual behavior should target both males and females, especially subgroups of students, who often consume alcohol in relation to sexual activity. Policy goals and programs should also aim to restrict the availability of alcohol and reduce its consumption on Ugandan university campuses.

## Competing interests

The authors declare that they have no competing interest.

## Authors’ contributions

VC contributed to the study design, conducted the statistical analysis, analysed the data and drafted the manuscript. POO contributed to the study design, data analysis, the protocol, interpretation of results and in the writing of the manuscript. AA developed the protocol, collected the data and contributed to the study design, the data analysis and to the writing of the manuscript. MS contributed to the data analysis and in the writing of the manuscript. All authors read and approve the final manuscript.

## Pre-publication history

The pre-publication history for this paper can be accessed here:

http://www.biomedcentral.com/1471-2458/14/128/prepub
